# ADAMTS2 Modulates Inflammation, Autophagy, and Barrier Integrity Through PI3K/AKT/mTOR Signaling in LPS‐Induced Acute Respiratory Distress Syndrome

**DOI:** 10.1002/iid3.70429

**Published:** 2026-04-20

**Authors:** Song Gao, Ting Lyu, Youhong Quan, Yaling Tao, Wenjin Wang, Xi Xu

**Affiliations:** ^1^ Department of Intensive Care Unit Xishan People's Hospital of Wuxi City (Wuxi Branch of Zhongda Hospital Southeast University) Wuxi China

**Keywords:** ADAMTS2, ARDS, autophagy, inflammation, PI3K/AKT/mTOR, tight junctions

## Abstract

**Background:**

Excessive inflammation and alveolar barrier dysfunction are commonly observed in acute respiratory distress syndrome (ARDS). ADAMTS2 is an ECM‐remodeling metalloproteinase with emerging immunomodulatory roles and may influence inflammatory injury and epithelial barrier stability via PI3K/AKT/mTOR signaling.

**Methods:**

Transcriptome sequencing of peripheral blood samples from ARDS patients revealed elevated ADAMTS2 expression. An in vitro ARDS model was established by stimulating human type II alveolar epithelial cells with LPS. The impact of ADAMTS2 silencing on inflammatory response, autophagic activity, tight junction proteins, and PI3K/AKT/mTOR signaling was examined through qRT‐PCR, Western blot, immunofluorescence, ELISA, and flow cytometry analyses. A rescue experiment was performed using the PI3K activator 740 Y‐P.

**Results:**

ADAMTS2 was significantly upregulated in ARDS transcriptomic analysis, with functional enrichment highlighting ECM‐related processes and PI3K/AKT/mTOR signaling. In LPS‐stimulated alveolar epithelial cells, ADAMTS2 expression was ~2.5–3.0‐fold higher than that in control cells. ADAMTS2 silencing reduced TNF‐α and IL‐1β release by ~55%–60% and decreased apoptosis by ~30%, while improving barrier integrity (increased ZO‐1/Occludin levels and TEER). Autophagy‐related changes were observed upon ADAMTS2 knockdown, including increased Beclin‐1 and LC3B‐II levels with reduced p62/SQSTM1 accumulation. Mechanistically, ADAMTS2 knockdown attenuated PI3K/AKT/mTOR phosphorylation, and these protective effects were partially reversed by the PI3K agonist 740 Y‐P.

**Conclusion:**

ADAMTS2 knockdown mitigated LPS‐induced epithelial injury and barrier dysfunction, in association with PI3K/AKT/mTOR signaling and autophagy‐related changes, suggesting ADAMTS2 as a potential therapeutic target for ARDS pending further in vivo validation.

## 1 Introduction

Acute respiratory distress syndrome (ARDS) is a critical condition resulting from both direct and indirect lung injuries such as pneumonia, aspiration, sepsis, and trauma [1]. Clinically, ARDS presents with sudden‐onset respiratory insufficiency, unresponsive low blood oxygen levels, and diffuse infiltrates in both lungs. Damage to the alveolar–capillary barrier results in elevated permeability, alveolar edema, and impaired gas exchange [2]. The LUNG‐SAFE study reported that in the ICU population, 10.4% satisfied the clinical definition of ARDS, with a 23.4% incidence among mechanically ventilated patients and a mortality rate near 40% [3]. The incidence of ARDS has significantly increased following the onset of the COVID‐19 pandemic, with ~32.2% of hospitalized patients developing ARDS and mortality rates reaching 40%–45% [4]. Despite advances in supportive care, effective targeted therapies are lacking, and the underlying mechanisms remain incompletely understood. Therefore, elucidating its molecular pathogenesis is of great significance for identifying novel therapeutic targets.

Among the various pathogenic mechanisms of ARDS, dysregulated inflammatory responses critically contribute to disease initiation and advancement [5]. Lipopolysaccharide (LPS) and other pathogen‐associated molecular patterns (PAMPs) initiate innate immune responses and provoke excessive cytokine release, ultimately compromising the alveolar–capillary interface, inducing pulmonary edema, and causing hypoxemia [6]. Proteins critical to tight junction (TJ) integrity, such as ZO‐1 and Occludin, are critical for epithelial barrier integrity, and their downregulation is closely associated with increased permeability [7]. In recent years, autophagy has emerged as an important regulator in ARDS [8]. By regulating inflammatory signaling and cell survival programs, autophagy can influence epithelial barrier stability, including TJ protein maintenance, under injurious conditions [9, 10]. Altered autophagy has also been observed in LPS‐induced acute lung injury/ARDS models [11, 12], supporting its relevance to early epithelial injury and barrier dysfunction.

The PI3K/AKT/mTOR axis is widely recognized for its role in modulating autophagy, inflammation, and epithelial barrier stability [13]. In ARDS models, aberrant activation of PI3K/AKT/mTOR suppresses autophagy and exacerbates inflammatory injury, whereas its inhibition restores autophagic activity and alleviates cellular injury [14]. Moreover, this pathway is closely associated with the expression of TJ proteins in alveolar epithelial cells, and its aberrant activation may lead to TJ protein downregulation and barrier dysfunction [15]. Therefore, intervening in the PI3K/AKT/mTOR cascade holds potential as an effective strategy to counteract ARDS development.

A disintegrin and metalloproteinase with thrombospondin motifs 2 (ADAMTS2) is classified as a crucial component of the ADAMTS protease family, primarily involved in extracellular matrix (ECM) remodeling by cleaving the N‐terminal propeptides of type I, II, and III procollagens [16]. Recent studies suggest that ADAMTS2 also plays important roles in inflammation and tissue repair [17, 18]. In patients with systemic lupus erythematosus, ADAMTS2 expression is significantly elevated in peripheral blood mononuclear cells and has been linked to activation of the TNFα–NF‐κB signaling cascade, suggesting its involvement in immune‐driven inflammation [19]. Although its function in ARDS remains unclear, glucocorticoid‐induced expression of ADAMTS2 in lung macrophages suggests potential involvement in pulmonary inflammation [20]. Notably, ADAMTS2^−/−^ mice exhibit abnormal lung structure with reduced parenchymal density, supporting a role for ADAMTS2 in pulmonary structural homeostasis [16]. In the injured lung, ECM remodeling shapes fibroblast activation, tissue repair, and epithelial barrier [21]. As ECM‐integrin signaling is an established upstream regulator of PI3K/AKT, ECM remodeling can activate integrin‐PI3K/AKT [22]. Therefore, the ECM‐modifying protease ADAMTS2 may plausibly influence PI3K/AKT/mTOR activity by reshaping the extracellular microenvironment. Notably, ADAMTS2 has been reported to be linked to PI3K/AKT‐dependent signaling in non‐pulmonary contexts [23]. However, whether ADAMTS2 directly regulates PI3K/AKT/mTOR signaling in the context of ARDS remains to be determined.

Building on this evidence, in this study, a cellular model of ARDS was developed by exposing human type II alveolar epithelial cells to LPS. The role of ADAMTS2 in modulating inflammation, autophagy, tight junction protein levels, and the PI3K/AKT/mTOR pathway was then comprehensively investigated. The findings may offer novel perspectives on alveolar barrier regulation and support the development of early molecular interventions and targeted therapies for ARDS.

## 2 Materials and Methods

### 2.1 RNA Sequencing and Bioinformatic Analysis

Peripheral whole blood samples were obtained from 6 healthy individuals, 13 patients with active ARDS, and 4 patients in the post‐treatment phase of ARDS. Total RNA was extracted and subjected to transcriptome sequencing by a commercial service provider (Honsunbio, China) using the Illumina NovaPE150 platform with a stranded mRNA‐seq protocol.

Gene expression profiling and statistical evaluation of differential expression were performed via the DESeq.2 package in R. Genes with an absolute log₂ fold change ≥ 1 and FDR‐adjusted *p* values < 0.05 were deemed significant. Functional analyses, encompassing GO and KEGG pathway enrichment, were undertaken using clusterProfiler. Data visualization, including volcano plots and heatmaps, was performed with ggplot2 and pheatmap in R.

### 2.2 Cell Culture and Treatment

Human type II alveolar epithelial cells (icell Bioscience, China) were cultured in primary epithelial cell culture medium (icell Bioscience) supplemented with 10% fetal bovine serum (FBS; Yeasen, China) and 1% penicillin‐streptomycin (Idraft, China), and maintained at 37°C in an incubator with 5% CO₂. Cells were used at passages 2–5 for all experiments to minimize passage‐related phenotypic changes.

To establish an in vitro ARDS model, cells were stimulated with 1 μg/mL lipopolysaccharide (LPS; Solarbio, China) for 24 h [24]; control cells received vehicle treatment alone (PBS) under identical conditions. ADAMTS2 expression was downregulated using three siRNAs (si‐ADAMTS2#1, #2, #3), with si‐NC serving as the negative control. Transfections were carried out with Lipofectamine 3000, and gene silencing efficacy was confirmed through qRT‐PCR and Western blotting.

All siRNAs were synthesized by Genscript (China). The guide strand sequences (5′−3′) were as follows:

si‐ADAMTS2#1: UCCUUUAAAACAAUGCUUGCC;

si‐ADAMTS2#2: ACAUCUUGAUGUAACCAUGCU;

si‐ADAMTS2#3: UCAAACAUCUUGAUGUAACCA;

si‐NC: UUCUCCGAACGUGUCACGUTT.

### 2.3 Quantitative Real‐Time PCR (qRT‐PCR)

Total RNA (1 μg) was reverse‐transcribed into cDNA using a commercial kit (Vazyme, China). Quantitative PCR was subsequently carried out on a QuantStudio 6 Flex system (Applied Biosystems, USA) employing SYBR Green Master Mix (Vazyme).

Primer sequences specific for human ADAMTS2 (NM_014244.4) were as follows: Forward: 5′‑TACAAGGACGCCTTCAGCCTCT‑3′; Reverse: 5′‑CCACTTTGCAGTGGCTGTTGTC‑3′. Relative gene expression levels were calculated using the 2^−ΔΔCt^ method, with GAPDH serving as an internal reference.

### 2.4 Western Blotting

Proteins were extracted with RIPA buffer (Absin, China) containing protease and phosphatase inhibitors. After SDS‐PAGE and transfer onto PVDF membranes, the blots were treated with blocking buffer and then probed with primary antibodies followed by HRP‐conjugated secondary antibodies. Detection was carried out using ECL (Univ‐bio, China), and band intensities were measured using ImageJ. Antibody sources and details are summarized in Table 1.

**Table 1:** Detailed information on antibodies used for Western blotting.

Antibody name	Host species	Dilution ratio	Catalog number	Manufacturer
ADAMTS2	Rabbit	1:1000	ab187839	Abcam
Occludin	Rabbit	1:1000	27260‐1‐AP	Proteintech
ZO‐1	Rabbit	1:1000	21773‐1‐AP	Proteintech
LC3B	Rabbit	1:1000	3868S	Cell signaling technology (CST)
Beclin‐1	Rabbit	1:1000	ab207612	Abcam
p62/SQSTM1	Rabbit	1:1000	ab109012	Abcam
PI3K (p85)	Rabbit	1:1000	4257S	CST
p‐PI3K (Tyr458)	Rabbit	1:1000	4228S	CST
AKT	Rabbit	1:1000	9272S	CST
p‐AKT (Ser473)	Rabbit	1:1000	9271S	CST
mTOR	Rabbit	1:1000	2983S	CST
p‐mTOR (Ser2448)	Rabbit	1:1000	5536S	CST
β‐actin	Mouse	1:5000	66009‐1‐Ig	Proteintech
HRP‐conjugated goat anti‐rabbit IgG	Goat	1:5000	SA00001‐2	Proteintech
HRP‐conjugated goat anti‐mouse IgG	Goat	1:5000	SA00001‐1	Proteintech

### 2.5 ELISA

The concentrations of TNF‐α and IL‐1β in cell supernatants were quantified using ELISA kits (Westang, China) following the supplier's protocol.

### 2.6 Immunofluorescence Staining

Sterile glass coverslips were used to culture cells prior to treatment application. Post‐treatment, they were immobilized using 4% paraformaldehyde, permeabilized with 0.2% Triton X‐100, and subsequently blocked with 5% BSA. LC3B immunostaining was performed via overnight incubation at 4°C with a specific primary antibody (CST, #3868, 1:200), followed by an Alexa Fluor 488‐labeled secondary antibody (Invitrogen, #A11008, 1:500). Nuclei were visualized using DAPI staining (Solarbio, China), and fluorescence signals were acquired using an Olympus IX73 microscope (Japan).

### 2.7 Cell Viability Assay

Cell viability was assessed using a Cell Counting Kit‐8 (CCK‐8, Beyotime, China). Briefly, alveolar epithelial cells were seeded into 96‐well plates and subjected to the indicated treatments. After treatment, 10 μL of CCK‐8 solution was added to each well and incubated for 2 h at 37°C. Absorbance was measured at 450 nm using a microplate reader.

### 2.8 Flow Cytometry

Cell apoptosis was evaluated using Annexin V‐FITC and propidium iodide staining kit (Westang). Cells were stained according to the kit protocol and analyzed using a flow cytometer (NovoCyte, Agilent, USA). Data were analyzed using NovoExpress software.

## 3 Transepithelial Electrical Resistance (TEER) Measurement

TEER was measured using an epithelial voltohmmeter (EVOM2, USA) to assess barrier function. Alveolar epithelial cells were cultured on Transwell inserts until a stable monolayer was formed and then subjected to the indicated treatments. TEER values were corrected by subtracting the resistance of blank inserts and normalized to the surface area.

### 3.1 Rescue Experiment With PI3K Pathway Activation

To determine if the PI3K/AKT/mTOR axis is involved in the regulatory effects of ADAMTS2 silencing, cells transfected with si‐ADAMTS2 were treated with the PI3K activator 740 Y‐P (20 μM; MedChemExpress, USA) 1 h prior to LPS exposure. Subsequent changes in autophagy, inflammatory cytokine secretion, TJ protein expression, and phosphorylation of signaling proteins were evaluated as described above.

### 3.2 Statistical Analysis

Each experiment was independently repeated three times. Data are presented as the mean ± standard deviation and were analyzed using GraphPad Prism version 9.0. Unpaired *t*‐tests or ANOVA with Tukey's test were used for normally distributed data, while Mann–Whitney U or Kruskal–Wallis tests were applied otherwise. A *p* value below 0.05 was regarded as statistically significant.

## 4 Results

### 4.1 ADAMTS2 Is Upregulated in ARDS and Enriched in Key Pathways

**Figure 1:**
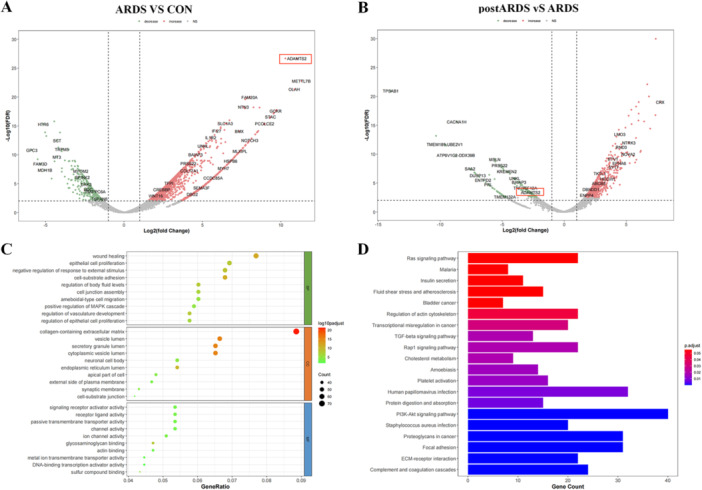
Transcriptomic analysis reveals elevated ADAMTS2 expression and pathway enrichment in ARDS. (A) Volcano plot of DEGs between ARDS and controls. (B) Volcano plot of DEGs between ARDS and post‐treatment ARDS groups. (C) GO enrichment analysis of DEGs. (D) KEGG analysis highlighting PI3K/AKT pathway involvement.

To explore the potential role of ADAMTS2 in ARDS, transcriptomic profiling of peripheral whole blood samples was performed. RNA sequencing revealed that Patients with ARDS exhibited markedly higher ADAMTS2 expression levels than healthy individuals, and its expression level declined in post‐treatment ARDS samples (Figure 1A,B). Functional enrichment analysis revealed that the differentially expressed genes (DEGs) were significantly associated with biological processes such as cell junction assembly and were predominantly enriched in the PI3K‐AKT signaling pathway, as shown in Figure 1C,D. These observations indicate a potential involvement of ADAMTS2 in controlling inflammation and maintaining epithelial barrier stability during ARDS.

### 4.2 ADAMTS2 Knockdown Attenuates LPS‐Induced Inflammation and Enhances Autophagy

**Figure 2:**
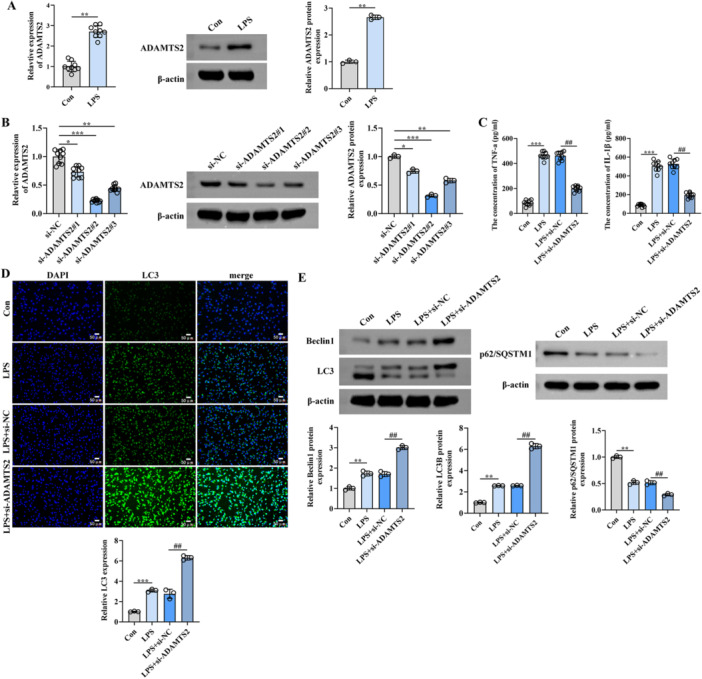
ADAMTS2 knockdown reduces inflammation and enhances autophagy in LPS‐stimulated A549 cells. (A) qRT‐PCR and Western blot showing ADAMTS2 upregulation after LPS stimulation. (B) Validation of siRNA knockdown efficiency. (C) ELISA showing decreased TNF‐α and IL‐1β after ADAMTS2 silencing. (D) LC3 puncta formation visualized by immunofluorescence. Original magnification, ×400. Scale bar = 100 μm. (E) Western blot analysis of Beclin1, LC3B, and p62/SQSTM1 indicating enhanced autophagy. *p < 0.05, **p < 0.01, ***p < 0.001; ^##^ *p* < 0.01.

To elucidate the role of ADAMTS2 in LPS‐induced alveolar epithelial injury, human type II alveolar epithelial cells were exposed to LPS, establishing an in vitro injury model. As shown in Figure 2A, ADAMTS2 expression was markedly upregulated upon LPS exposure. To explore its functional significance, three siRNAs were synthesized for knocking down ADAMTS2. Among them, si‐ADAMTS2#2 exhibited the most potent suppression at both mRNA and protein levels and was thus selected for subsequent experiments (Figure 2B). ELISA assays demonstrated that ADAMTS2 knockdown notably suppressed the secretion of representative cytokines TNF‐α and IL‐1β in LPS‐stimulated cells compared with the LPS + si‐NC group, suggesting an anti‐inflammatory effect (Figure 2C). Furthermore, immunofluorescence staining showed that si‐ADAMTS2 increased LC3 puncta formation, indicative of enhanced autophagy (Figure 2D). This finding was further supported by Western blot results, which showed increased Beclin‐1 and LC3B‐II levels together with reduced p62/SQSTM1 accumulation following ADAMTS2 silencing (Figure 2E). Together, the results provide evidence supporting that ADAMTS2 knockdown attenuates LPS‐induced inflammation and promotes autophagy activation in alveolar epithelial cells.

### 4.3 ADAMTS2 Knockdown Mitigates LPS‐Induced Epithelial Injury and Barrier Dysfunction in Alveolar Epithelial Cells

**Figure 3:**
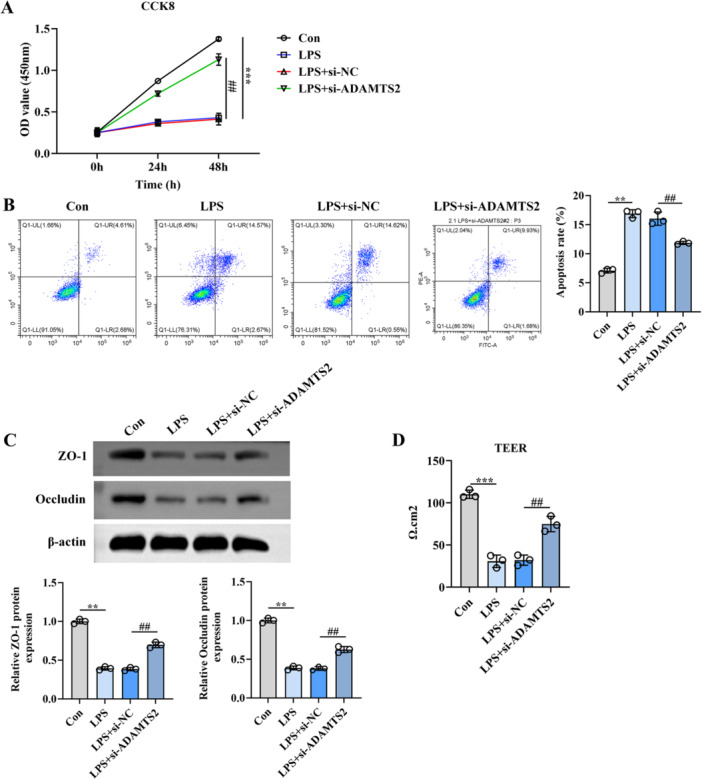
Silencing ADAMTS2 attenuates LPS‐induced epithelial injury and barrier disruption in alveolar epithelial cells. (A) Cell viability was assessed by CCK‐8 assay; (B) flow cytometry analysis of apoptosis. (C) Western blot analysis of Occludin and ZO‐1. (D) TEER measurements of epithelial barrier function.**p < 0.01, ***p < 0.001, ^##^ *p* < 0.01.

To gain deeper insight into the involvement of ADAMTS2 in regulating epithelial barrier integrity and apoptosis, we assessed cell viability, apoptotic rates, TJ protein levels, and barrier function following LPS exposure. CCK‐8 assays showed that LPS (1 µg/mL, 24 h) significantly reduced cell viability, whereas ADAMTS2 silencing partially restored viability (Figure 3A). An elevated apoptotic cell population was observed in the LPS and LPS + si‐NC groups, as determined by flow cytometry, while ADAMTS2 silencing significantly reduced the apoptosis rate (Figure 3B). Occludin and ZO‐1 expression levels were markedly downregulated upon LPS exposure, as demonstrated by Western blotting, relative to the control group. However, knockdown of ADAMTS2 notably restored the expression of these TJ proteins (Figure 3C). Consistently, TEER measurements revealed a pronounced decrease in barrier function after LPS stimulation, which was significantly attenuated by ADAMTS2 knockdown (Figure 3D). These findings suggest that silencing ADAMTS2 protects against LPS‐induced TJ disruption and apoptosis in alveolar epithelial cells.

### 4.4 ADAMTS2 Knockdown Inhibits LPS‐Induced Activation of the PI3K/AKT/mTOR

**Figure 4:**
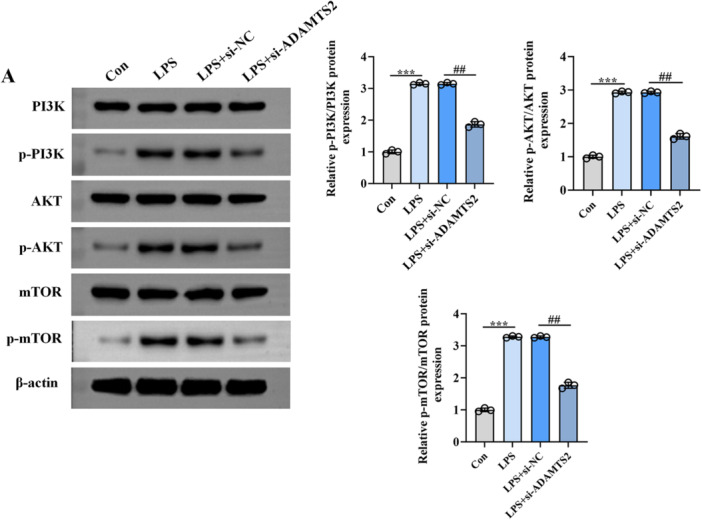
ADAMTS2 knockdown attenuates LPS‐induced activation of the PI3K/AKT/mTOR signaling pathway. (A), Western blot analysis of total and phosphorylated PI3K, AKT, and mTOR in control, LPS, LPS + si‐NC, and LPS + si‐ADAMTS2 groups. β‐actin was used as a loading control. ***p < 0.001, ^##^ *p* < 0.01.

To explore the regulatory effect of ADAMTS2 on the PI3K/AKT/mTOR axis in LPS‐stimulated alveolar epithelial cells, total and phosphorylated forms of PI3K, AKT, and mTOR were evaluated by Western blotting. As shown in Figure 4, LPS exposure led to enhanced phosphorylation of PI3K, AKT, and mTOR relative to the control group, indicating activation of this pathway. This effect was not altered by si‐NC transfection. However, knockdown of ADAMTS2 (LPS + si‐ADAMTS2 group) markedly reduced the phosphorylation levels of all three proteins, while the total protein levels remained unchanged across groups. Taken together, these data indicate that ADAMTS2 knockdown is associated with reduced PI3K/AKT/mTOR phosphorylation under LPS stimulation, supporting a role for ADAMTS2 in the modulation of this pathway.

### 4.5 PI3K/AKT/mTOR Pathway Activation Reverses the Protective Effects of ADAMTS2 Knockdown

**Figure 5:**
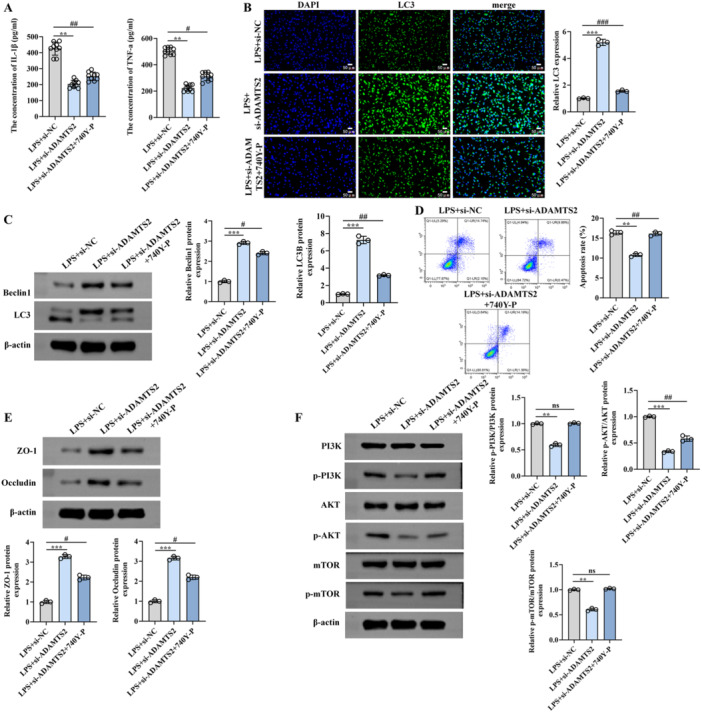
PI3K/AKT/mTOR pathway activation reverses the protective effects of ADAMTS2 knockdown. 740 Y‐P treatment restores inflammatory cytokine levels (A), reduces autophagy (B, C), increases apoptosis (D), decreases TJ proteins (E), and reactivates PI3K/AKT/mTOR signaling (F). Original magnification of (B), ×400. Scale bar = 100 μm. **p < 0.01, ***p < 0.001; ^#^ *p* < 0.05, ^##^ *p* < 0.01; ns = no significant.

To confirm whether the protective effects of ADAMTS2 silencing were mediated through suppression of PI3K/AKT/mTOR pathway, si‐ADAMTS2‐transfected cells were pretreated with the PI3K activator 740 Y‐P before LPS exposure. ELISA results showed that knockdown of ADAMTS2 significantly reduced the levels of representative cytokines TNF‐α and IL‐1β, which were partially restored upon 740 Y‐P treatment (Figure 5A). Immunofluorescence analysis of LC3 and Western blotting for Beclin1, LC3B, and p62/SQSTM1 showed that ADAMTS2 silencing increased LC3B/Beclin‐1 levels while reducing p62 accumulation, and these effects were attenuated by 740 Y‐P treatment (Figure 5B,C). Flow cytometry indicated that the reduction in LPS‐induced apoptosis by si‐ADAMTS2 was reversed by 740 Y‐P (Figure 5D). In terms of epithelial barrier function, knockdown of ADAMTS2 increased ZO‐1 and Occludin expression, whereas 740 Y‐P diminished this effect (Figure 5E). Consistently, TEER measurements demonstrated that the barrier‐protective effect conferred by ADAMTS2 silencing was significantly abrogated by PI3K activation (Figure 5F). Finally, phosphorylation levels of PI3K, AKT, and mTOR, which were suppressed by ADAMTS2 silencing, were restored upon 740 Y‐P treatment, as shown by Western blot analysis (Figure 5F). Together, these results support the involvement of PI3K/AKT/mTOR signaling in the cellular response to ADAMTS2 knockdown in LPS‐exposed alveolar epithelial cells.

## 5 Discussion

In this study, we identified ADAMTS2 as a previously unrecognized contributor to the pathogenesis of ARDS. Transcriptomic analysis demonstrated a marked upregulation of ADAMTS2 in the peripheral blood of ARDS patients, accompanied by the enrichment of inflammatory and autophagy‐related pathways. Functional validation using an in vitro model of LPS‐induced epithelial injury revealed that silencing ADAMTS2 attenuated the production of inflammatory factors, promoted autophagy, preserved tight junction integrity, and reduced apoptosis. These protective effects were linked to the inhibition of the PI3K/AKT/mTOR signaling cascade, suggesting that ADAMTS2 may act upstream to regulate this critical pathway during ARDS progression.

Beyond its well‐characterized role in ECM dynamics, emerging evidence suggests that ADAMTS2 may also participate in immune modulation and inflammatory signaling [25, 26]. Building on these findings, our study sheds new light on the previously underexplored involvement of ADAMTS2 in the development of acute lung injury. Specifically, we demonstrate that ADAMTS2 expression is markedly upregulated in both peripheral blood samples from ARDS patients and in LPS‐induced alveolar epithelial injury models. This increase coincides with heightened inflammatory responses and epithelial barrier disruption, key features of ARDS pathogenesis [27]. These findings underscore the functional involvement of ADAMTS2 in the progression of lung injury and point to its potential as a diagnostic indicator or therapeutic intervention target in ARDS.

Because ADAMTS2 directly participates in ECM remodeling, the inflammatory phenotypes observed in our study may, at least in part, arise from altered ECM–cell interactions. Changes in ECM homeostasis are known to influence integrin‐mediated signaling, which can activate FAK and downstream PI3K/Akt pathways [28]. In parallel, ECM remodeling can generate matrix‐derived danger signals that act as DAMPs and amplify innate inflammatory responses via pattern‐recognition receptors, including TLRs [29]. Moreover, changes in pericellular matrix architecture can shape immune cell recruitment and migration within injured tissues, thereby influencing the inflammatory milieu [30]. While these upstream links were not directly interrogated in the present study, they provide a coherent mechanistic framework linking ADAMTS2‐mediated ECM remodeling to inflammatory regulation and PI3K/AKT/mTOR‐dependent cellular responses in ARDS.

Notably, autophagy plays context‐dependent roles in ARDS, exerting both protective and detrimental effects depending on disease stage and severity [31]. Under moderate stress, by promoting the removal of dysfunctional organelles, protective autophagy plays a vital role in preserving epithelial barrier function [32, 33]. However, contextual variability significantly influences the functional consequences of autophagy in ARDS, as shown in recent research. For instance, Wen et al. reported that insulin therapy restored alveolar fluid clearance by suppressing LPS‐triggered autophagy and inflammatory responses, implicating excessive autophagy as a detrimental factor in septic lung injury [8]. In contrast, Wei et al. reported that activation of autophagy by SIRT6 alleviated LPS‐induced epithelial apoptosis and TJ disruption via the ERK1/2 pathway, indicating a cytoprotective role for autophagy under certain regulatory conditions [9]. Our findings further support the protective aspect of autophagy in the setting of ARDS. Specifically, knockdown of ADAMTS2 significantly upregulated autophagy‐related proteins such as LC3B and Beclin‐1, accompanied by reduced p62/SQSTM1 accumulation. These changes were paralleled by decreased activation of mTOR, a key inhibitor of autophagy, supporting the hypothesis that ADAMTS2 knockdown promotes autophagy‐related protective changes by inhibiting the PI3K/AKT/mTOR pathway.

Furthermore, knockdown of ADAMTS2 preserved TJ proteins ZO‐1 and Occludin, indicating enhanced epithelial barrier integrity, which was further supported by improved TEER measurements. TJ disruption is a hallmark of ARDS, contributing to alveolar–capillary leakage, pulmonary edema, and respiratory failure [34]. Pro‐inflammatory cytokines and oxidative stress induced by LPS have been shown to downregulate or mislocalize TJ proteins [35, 36]. Our results suggest that ADAMTS2 may exacerbate TJ disruption under inflammatory conditions, whereas its silencing helps maintain junctional architecture, possibly by limiting inflammation and preserving cytoskeletal integrity. These findings reveal a novel role for ADAMTS2 in barrier regulation and support its potential as a therapeutic target to protect epithelial integrity in ARDS.

To our knowledge, this is the first study to implicate ADAMTS2 in regulating autophagy and epithelial barrier integrity in the context of ARDS. Despite these promising findings, several limitations should be noted. First, although primary human alveolar epithelial cells were used to better mimic the pulmonary epithelial environment, in vitro models cannot fully recapitulate the complexity of the ARDS microenvironment in vivo, and therefore require further validation in appropriate animal models and clinical settings. Second, although transcriptomic analysis revealed elevated ADAMTS2 expression in ARDS patients, the limited sample size, particularly in the post‐treatment ARDS group, limits the statistical robustness and generalizability of these findings; this limitation was mainly due to the practical and ethical challenges in obtaining paired post‐treatment blood samples from critically ill ARDS patients. In addition, the lack of spatial resolution in bulk RNA sequencing limits definitive identification of the cell‐type specificity and spatial distribution of ADAMTS2 in the lung. Third, the exact mechanism by which ADAMTS2 regulates the PI3K/AKT/mTOR pathway has not been fully elucidated, as the present mechanistic evidence is primarily derived from loss‐of‐function experiments and may involve indirect effects such as extracellular matrix remodeling or interaction with other signaling intermediates.

Collectively, our findings suggest that ADAMTS2 may be a potential modulator of epithelial injury in ARDS, and that its inhibition may serve as a potential strategy to alleviate inflammation and preserve barrier function through autophagy regulation.

## Author Contributions

**Song Gao** and **Ting Lyu:** conceptualization, methodology, data curation, writing – original draft, investigation, formal analysis, and visualization. **Hong Quanyou** and **Yaling Tao:** validation and supervision. **Wenjin Wang:** software. **Xi Xu:** conceptualization, methodology, writing – review and editing.
